# Chronic pulmonary fibrosis alters the functioning of the respiratory neural network

**DOI:** 10.3389/fphys.2023.1205924

**Published:** 2023-06-13

**Authors:** Céline-Hivda Yegen, Dominique Marchant, Jean-François Bernaudin, Carole Planes, Emilie Boncoeur, Nicolas Voituron

**Affiliations:** ^1^ Laboratoire Hypoxie & Poumon, UMR INSERM U1272, Université Sorbonne Paris Nord, Bobigny, France; ^2^ Faculté de Médecine, Sorbonne Université, Paris, France; ^3^ Service de Physiologie et d’Explorations Fonctionnelles, Hôpital Avicenne, APHP, Bobigny, France; ^4^ Département STAPS, Université Sorbonne Paris Nord, Bobigny, France

**Keywords:** lung injury, central respiratory drive, neuroplasticity, IPF—idiopathic pulmonary fibrosis, FOSB

## Abstract

Some patients with idiopathic pulmonary fibrosis present impaired ventilatory variables characterised by low forced vital capacity values associated with an increase in respiratory rate and a decrease in tidal volume which could be related to the increased pulmonary stiffness. The lung stiffness observed in pulmonary fibrosis may also have an effect on the functioning of the brainstem respiratory neural network, which could ultimately reinforce or accentuate ventilatory alterations. To this end, we sought to uncover the consequences of pulmonary fibrosis on ventilatory variables and how the modification of pulmonary rigidity could influence the functioning of the respiratory neuronal network. In a mouse model of pulmonary fibrosis obtained by 6 repeated intratracheal instillations of bleomycin (BLM), we first observed an increase in minute ventilation characterised by an increase in respiratory rate and tidal volume, a desaturation and a decrease in lung compliance. The changes in these ventilatory variables were correlated with the severity of the lung injury. The impact of lung fibrosis was also evaluated on the functioning of the medullary areas involved in the elaboration of the central respiratory drive. Thus, BLM-induced pulmonary fibrosis led to a change in the long-term activity of the medullary neuronal respiratory network, especially at the level of the nucleus of the solitary tract, the first central relay of the peripheral afferents, and the Pre-Bötzinger complex, the inspiratory rhythm generator. Our results showed that pulmonary fibrosis induced modifications not only of pulmonary architecture but also of central control of the respiratory neural network.

## Introduction

Idiopathic pulmonary fibrosis (IPF) is an interstitial lung disease characterised by an excessive collagen deposition in the distal lung leading to a fatal restrictive respiratory failure. In the absence of effective treatment, the median survival from diagnosis is around 3 years with a variable disease course among patients: slowly progressive, with occasional exacerbation episodes, or more rarely rapidly progressive (King et al., 2011; Raghu et al., 2011) leading to high mortality (Dempsey, 2006). Histologically, IPF is characterised by a patchy dense fibrosis with honeycomb predominating in the subpleural and paraseptal parenchyma and fibroblastic foci. Currently, the main pathophysiologic hypothesis consists of a deregulation of the dialog between fibroblasts and the alveolar epithelial cells composing the alveoli. This deregulation would play an essential role in the process of fibrosis development by allowing an aberrant healing of the alveolar epithelium following repeated alveolar micro-injuries, and excessive collagen deposition (Raghu et al., 2011; Bendstrup, 2014; 2014). The excess of collagen and the transformation of the parenchyma into scar tissue is at the origin of pulmonary stiffness in patients with IPF, decreasing lung volumes (Nava and Rubini, 1999) and finally results in a decline in lung function (Plantier et al., 2018). Pulmonary compliance is an important component of the mechanical load of respiratory muscles and the work of breathing (Crystal, 1997). Thus, ventilatory adaptations may occur to overcome these external mechanical loads. Indeed, dyspnea, impairment of ventilatory variables characterised by a low forced vital capacity associated with an increase in respiratory frequency and a decrease in tidal volume were found in patients with interstitial lung diseases (Javaheri and Sicilian, 1992). In addition to the mechanical effects related to changes in lung stiffness, alteration in pulmonary gas exchange is also observed in IPF patients who present a reduction of lung diffusing capacity, an increase in physiological dead space, and chronic arterial hypoxemia in the most severe forms of the disease (Plantier et al., 2018). Furthermore, at least in the resting condition, a ventilation-perfusion inequality was observed (Jernudd-Wilhelmsson et al., 1986; Agustí et al., 1991). It was recently suggested that alteration of lung compliance and impaired gas exchange could affect ventilatory drive (Plantier et al., 2018). Furthermore, the activity of the respiratory network being sensitive to peripheral afferents, IPF could affect the central respiratory drive through its consequences on ventilatory mechanics and/or gas exchange, leading to an increase in the command to the respiratory muscles (Van Meerhaeghe et al., 1981; Gaultier, 1983; Raux et al., 2007).

A ponto-medullary neuronal network elaborates the central respiratory command (Del Negro et al., 2018). Within this network, the Respiratory Rhythm Generators (RRG) produce the basic oscillatory activity of ventilation (Funk and Greer, 2013). In particular, the Pre-Bötzinger Complex is necessary and sufficient for the generation of inspiration (Anderson and Ramirez, 2017). Then, the Central Pattern Generator (CPG), usually described as a bilateral, mainly ventral, ponto-bulbar column, translates this basic oscillation into a coordinated pattern of activity, which is transmitted to the respiratory motoneurons (Funk and Greer, 2013). The CPG permanently receives a large number of peripheral and central afferents modulating its activity in order to adjust the ventilatory pattern to the metabolic (blood gases, pH) or mechanical (e.g., posture, lung inflation, tracheal pressure, thoracic expansion, etc.) information. The lung is a richly innervated organ. Retrograde tracing from the lung highlighted neurons primarily in the vagal ganglia which project specifically to the nucleus of the solitary tract in the brainstem (Su et al., 2022). Thus, mechanical afferents [Rapidly Adapting Receptors (RAR) or Slowly Adapting Receptors (SAR)] located in the lung parenchyma, bronchi and respiratory muscles (Raux et al., 2007), as well as chemosensitive afferents [peripheral and central chemoreceptors (Nattie and Li, 2012)] or C-type fibers in the airways and lungs are able to modulate pulmonary ventilation in response to various stimuli. The first central relay of information from these peripheral afferents corresponds to the commissural and medial subdivisions of the nucleus of the tractus solitarius (SolC and SolM), which are the main areas of projection of sensory fibres from the sinus nerve (Torrealba and Claps, 1988) and vagal nerve (Kalia and Mesulam, 1980; Contreras et al., 1982). Neurons in the SolC and SolM integrate and relay information from these peripheral afferents to other regions of the central nervous system involved in the elaboration of central respiratory control.

The neuronal respiratory network has a high level of plasticity (Mitchell and Johnson, 2003). This plasticity is defined by a persistent change in the neural control system induced by past experiences (Mitchell and Johnson, 2003). This phenomenon may involve structural and functional modifications at the neuronal respiratory network level. Indeed, as described above, rhythm generation and pattern formation are permanently influenced by chemoreceptors, mechanoreceptors and neuromodulatory systems (Mitchell and Johnson, 2003). Moreover, plasticity could be induced by hypoxia, hypercapnia or lung injury (Mitchell and Johnson, 2003). Indeed, numerous components of neuronal respiratory network can show neuroplasticity after lesion-induced changes in breathing behaviour (Forster, 2003).

In this context, our hypothesis was that damage to the lung parenchyma due to pulmonary fibrosis would lead to changes in the respiratory neuronal network though neuroplasticity mechanisms. Using a mouse model of chronic lung fibrosis induced by repeated instillations of BLM (Degryse et al., 2010; Boncoeur et al., 2022; Yegen et al., 2022), we investigated changes in the ventilatory mechanics as well as pulmonary fibrosis-induced neuronal plasticity at the respiratory network level.

## Methods

### Animals and ethical approval

As IPF is a prevalent disease in males (Raghu et al., 2011; Cottin et al., 2013; Caminati et al., 2015), all experiments were conducted in C57Bl/6j male mice (n = 12, Janvier Labs, Le Genest-Saint-Isle, France). Animals were aged about 8 weeks and weighed 23.5 ± 1.0 g at the beginning of experiments. The animals were housed in standard cages with a 12 h/12 h lighting conditions and received a standard diet with *ad libitum* access to drinking water in our animal facility (agreement number C9300801). After 1 week of acclimatization, experimental procedures were initiated.

### Induction of pulmonary fibrosis

**FIGURE 1:**
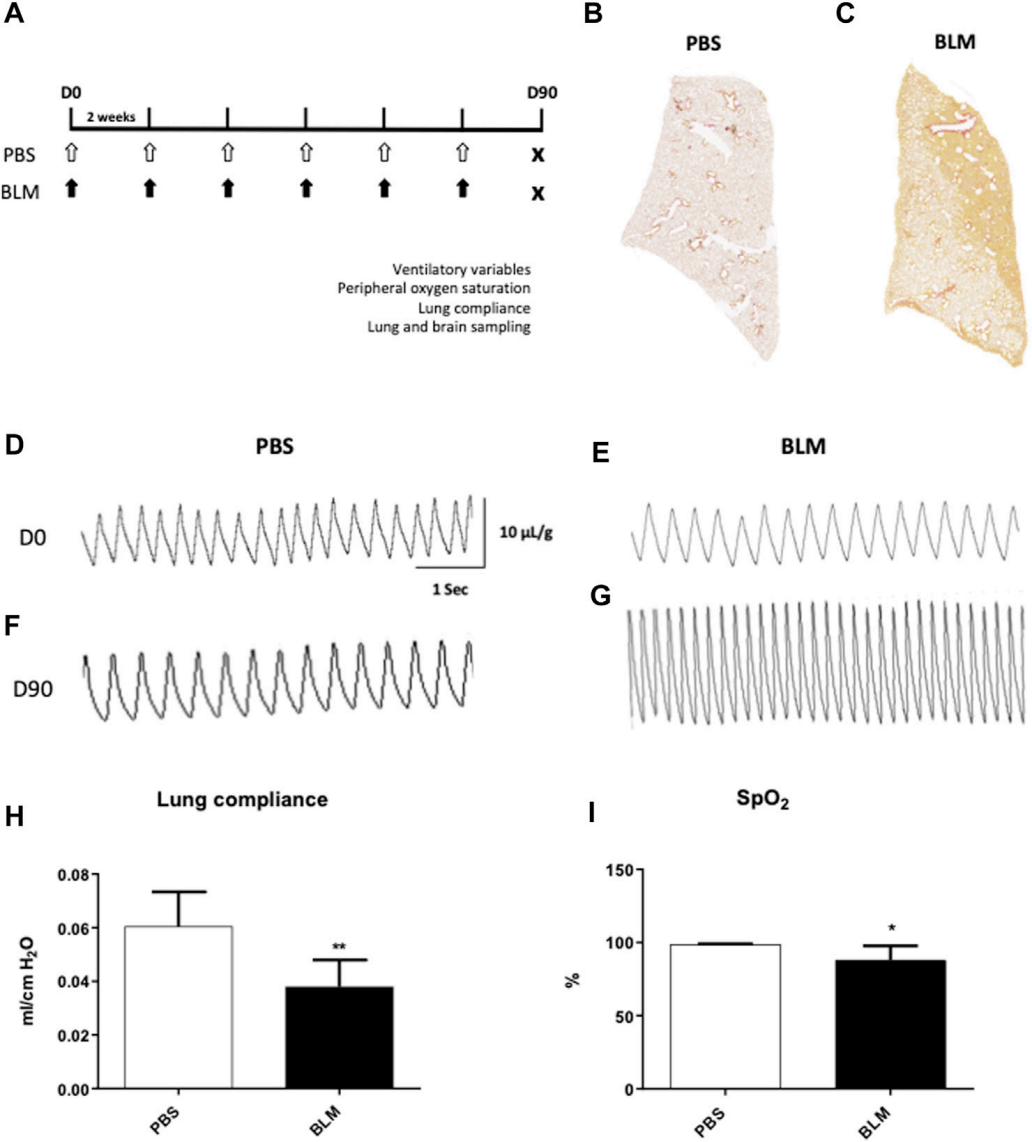
Measurement of ventilatory variables of mice developing pulmonary fibrosis obtained by repeated instillations of bleomycin. **(A)** Experimental protocol. Eight week-old C57BL6/J male mice received 12 intra-tracheal instillations of bleomycin (BLM, black arrows, *n* = 6) at 0.8 IU/g or PBS (PBS, white arrows, *n* = 6) every 2 weeks (Yegen et al., 2022). At day 90 (D90) ventilatory variables, peripheral oxygen saturation and respiratory system compliance were evaluated. After that, the mice were sacrificed and the lungs and brain were removed. **(B,C)** Cartography of a 5 µm thick section of lung stained with Sirius Red from PBS **(B)** and BLM **(C)** groups. A representative lung slice is shown for each group. **(D–G)** Typical plethysmographic recordings of breathing in PBS **(D,F)** or BLM **(E,G)** groups recorded at D0 **(D,E)** and at the end of experimental induction of pulmonary fibrosis at D90 **(F,G)**. Calibration bar in x-axis: 1 s and y-axis: 10 μL/g. **(H)** Measurement of lung compliance by covariance method (C_cov_, mL/cm H_2_O) using whole body plethysmography on anesthetized and tracheomized mice in the PBS (*n* = 6) and BLM (*n* = 6) groups. **(I)** Measurement of peripheral oxygen saturation (SpO_2_, %) by pulse oximeter in the PBS (*n* = 6) and BLM (*n* = 6) groups. All values were represented as mean ± SD. *p < 0.01, **p < 0.001.

As recently published (Yegen et al., 2022), we have developed an original model of pulmonary fibrosis in C57Bl/6j mice, obtained by repeated intra-tracheal instillations of BLM (Sigma-Merck, Saint-Quentin-Fallavier, France) under general anesthesia (3% isoflurane). Briefly, 6 intra-tracheal instillations of low doses of BLM (0.8 UI. g^-1^, *n* = 6) or PBS (*n* = 6) were performed with an interval of 2 weeks between two administrations (Figure 1A) (Boncoeur et al., 2022; Yegen et al., 2022). Survival and body weight were monitored during the entire experimental protocol (See Yegen et al., 2022 (Yegen et al., 2022) for more details). All the following analyses were carried out at day 90 (D90), 2 weeks after the last instillation (Figure 1A).

### *In vivo* measurement of ventilatory variables

As previously described (Voituron et al., 2009; Niane et al., 2011; Samillan et al., 2013; Jeton et al., 2022), breathing variables were measured non-invasively in unanesthetized and unrestrained animals using whole-body flow barometric plethysmograph (Emka technologies, Paris, France). This method consists of measuring the pressure variations in a recording chamber during spontaneous ventilation. Briefly, mice were placed in a recording chamber ventilated with air at room temperature (21°C–22°C) and the ventilatory variables were analysed through a differential pressure transducer that measured the pressure difference between the recording chamber and a reference chamber. Thus, whole body plethysmography provides a measurement of respiratory frequency (*f*_R_ in cycle per min, c. min^-1^), as well as tidal volume (V_T_ in µl) and minute ventilation ( $\dot{V}$ e in ml. min^-1^), which were normalized by the mice body weight (V_T_, μl g^-1^ and $\dot{V}$ e, ml.g^-1^. min^-1^). The Ti/Ttot and V_T_/Ti ratios were also determined to provide an indication of breathing time and an index of inspiratory drive (Milic-Emili and Grunstein, 1976).

### Peripheral oxygen saturation

After short sedation with isoflurane 3% during 2 min, animal neck was shaved and then a collar sensor was put in place. After waking, mice were habituated for 10 min and the peripheral blood saturation (SpO_2_, %) was recorded during 10 min in normoxic condition through a non-invasive infrared pulse oximetry (mouseOx Plus, Starr Life Science) (Lax et al., 2014; Gille et al., 2018).

### Evaluation of lung compliance

Pulmonary compliance was evaluated in mice using plethysmography (Emka technologies). Briefly, mice were sedated (ketamine/xylazine I.P. injection; 100 mg/kg and 20 mg/kg respectively), tracheotomised and ventilated (RoVent Jr., Kent Scientific Corporation; respiratory frequency = 150 bpm, Tidal Volume = 0.27 mL; Insp/Exp ratio = 0.40). A differential pressure transducer was used to obtain a flow signal, which reflects the expansion and contraction of the thorax during each ventilation cycle. Compliance and resistance were calculated following the acquisition of flow and pressure signals. Volume signal was obtained by integration of the flow signal measured by the differential pressure transducer. As the pneumotachograph was the only way for air to flow into and out of the chamber, the difference of pressure between inside and outside was proportional to this flow.

### Lung and brain sampling

After measuring lung compliance, a thoracotomy was performed. Then, right pulmonary lobes were isolated through a ligation to the hilum to prevent the passage of the perfusion solution. Mice were then transcardially perfused with 0.9% saline-buffered solution followed by 4% paraformaldehyde (Chem Cruz, SC-253236B) in 0.1 M Phosphate-buffered saline solution (pH 7.4). Thus, the left lobes as well as the brain were fixed. After fixation, the brain and the heart/lung unit were removed. The right lung lobes were separated, frozen in liquid nitrogen and stored at −80°C for other studies. The left lobes were placed in 4% paraformaldehyde for 24 h and paraffin embedded. Sections were cut at 5 µm thickness for Sirius Red staining. Finally, the brain was post-fixed for 48 h at 4°C and then cryoprotected in 30% sucrose solution for 48 h at 4°C for immunohistochemistry.

### Quantification of collagen

Lung sections of 5 µm thickness were dewaxed in two xylene (Carlo Erba, 392602) baths and then rehydrated by successive ethanol baths at decreasing concentration (absolute ethanol: VWR, 20820362). The collagen fibers 1 and 3 were stained with 0.1% Sirius Red (Sigma, Cat#365548) and washed with acidified water. Then, preparations were dehydrated with absolute alcohol, cleared with xylene and mounted with a hydrophobic mounting solution.

### Evaluation of the fibrosis score

Additional 5 µm paraffin sections of the left lung were used. After paraffin removal with xylene (Carlo Erba, 392602), the tissue was re-hydrated by successive baths of decreasing concentration of ethanol (absolute ethanol: VWR, 20820362), the alveolar epithelium was counterstained with 0.1% Fast Green (Merck, F7252) and type 1 and 3 collagen fibers were stained with 0.1% Sirius Red (Sigma, Cat#365548). Subsequently, the sections were dehydrated, mounted and observed under the optic microscope. To quantify the severity of fibrosis, we developed a new score derived from the Aschroft score with a panel of five images ranging from an image of a healthy lung with a normal lung without architectural remodeling (score 1) to a completely remodeled lung (score 5) which describes the major remodeling observed in pulmonary fibrosis (Ashcroft et al., 1988). The score 1 corresponds to a lung with no fibrotic burden at the most flimsy small fibers in some alveolar walls and with normal lung. Score 2 correspond to clearly fibrotic changes with alveoli partly enlarged and rarefied, but no fibrotic masses. Score 3 correspond to presence of single fibrotic masses (>10% of microscopic field). Score 4 correspond to lung with confluent fibrotic masses (>10%) with a lung structure severely damaged but still preserved and score 5 correspond to large continuous fibrotic masses (>50%) and lung architecture mostly not preserved.

### Immunohistochemistry

#### Analysis of FOSB/ΔFOSB-positive cells expression

The pulmonary fibrosis-induced neuroplasticity was assessed by immunodetection of FOSB/ΔFOSB in cytoplasm and nucleus, a long-term neuronal marker (Baum et al., 2018). Indeed, FOSB, especially the n-terminal truncated form is very stable and resists well to degradation. The brainstem was coronally sectioned at 40 µm using a cryostat. One section out of two was collected in 0.1 M Phosphate-buffered saline solutions for immunohistochemistry processing. The other ones were placed in cryoprotective solution and stored at −20°C for later use. Sections were processed for FOSB/ΔFOSB immunohistochemistry as previously described (Perrin-Terrin et al., 2016; Baum et al., 2018). Sections were incubated for 48 h at 4°C with a mouse monoclonal antibody directed against FOSB/ΔFOSB (F-7, a mouse monoclonal antibody, Sc-398595 Santa Cruz, used at 1:2000). Then, sections were incubated 2 h at room temperature with a biotinylated goat anti-mouse secondary antibody (Vector Laboratories BA200 used at 1:2000) and for 1 h with avidin-biotin-peroxydase complex (ABC, VECTASTAN, Elit PK-100 standard, ZE0622). Peroxydase activities were detected using 0.015% 3.3′-diaminobenzidine tetrahydrochloride (Roth, CN75.3), 0.4% nickel ammonium sulphate (Alfa Aesar, 12519), and 0.006% hydrogen peroxide (Fischer BioReagents, BP2633) in 0.2 M tris-HCl buffer (pH 7.6). Sections were washed, mounted on silane-treated slides, air-dried, dehydrated with absolute alcohol, cleared with xylene and coverslipped. Control sections were treated in parallel without primary or secondary antibodies. No labelling was observed in these conditions.

Sections were examined under a light microscope (Axioskop Zeiss Germany) and FOSB/ΔFOSB-positive cells were analysed in brainstem structures related to respiratory control using standard landmarks (Paxinos et al., 2001): commissural part, median part and ventrolateral part of nucleus of the solitary tract (SolC, SolM and SolVL respectively); *raphe magnus nucleus* (*RMg*); *raphe pallidus nucleus* (*RP*a); *raphe obscurus nucleus* (*ROb*); retrotrapezoid nucleus/parafacial respiratory group (RTN/pFRG) and hypoglossal nucleus (12N). Counts of FOSB/ΔFOSB-positive cells were performed at higher magnification (×400). Bilateral structures were analysed on one side and positive cells were counted in the entire area for the median structures. For each structure, results were expressed as the mean number of positive cells per section. Distribution of FOSB/ΔFOSB-positive cells was reported on representative drawings adapted from the Paxinos atlas (Paxinos et al., 2001) to illustrate their location.

#### Double labelling of FOSB/ΔFOSB-positive cells with neurokinin 1 receptor (NK1-R) or serotonin (5-HT)

The distribution analysis of FOSB/ΔFOSB-positive cells was associated with the one of Neurokinin 1 receptor (NK1-R), a G-protein-coupled receptor located on the cell membrane. NK1-R staining was used to localise more precisely the inspiratory rhythm generator [the Pre-Bötzinger complex, (Gray et al., 2001)]. Dual labelling with serotonin (5-HT) was also realized in order to investigate the serotoninergic character of the FOSB/ΔFOSB-positive cells at the raphe nuclei level. Indeed, serotoninergic systems are known to be involved in many processes of ventilatory neuroplasticity (Baum et al., 2018). For this, FOSB/ΔFOSB staining was realized according to the same protocol as described above. Then sections were incubated with NK1-R antibody (Anti-substance P receptor antibody produced in rabbit, Sigma Aldrich S8305, 1:2500) or 5-HT antibody (Anti-Serotonin antibody produced in rabbit, Sigma Aldrich S5545, 1:2500) for 48 h at 4°C followed by incubation with secondary biotinylated antibody (Biotinylated Anti rabbit IgG produce in horse, Vector Laboratories, BA-1100 1:2000) for 2 h at room temperature and then for 1 h with ABC kit. Thereafter, NK1-R labelling or 5-HT labelling was detected with 0.192% DAB (Roth, CN75.3), 3.6% NaCl (Fisher S/3160/60), 4% Nickel (Alfa Aesar 12519) and 0.0125% H_2_O_2_. Sections were then washed, mounted on silane-treated slides, air-dried, dehydrated with absolute alcohol, cleared with xylene and coverslipped. Control sections were made without primary or secondary antibodies. No labelling was observed in these conditions.

The sections analysis was performed as described above. Counts of FOSB/ΔFOSB-positive cells were performed at higher magnification (×400) and results were expressed as the mean percentage of double-labelled cells among the total number of FOSB/ΔFOSB-positive cells in the Pre-Bötzinger complex (NK1-R) or the medullary Raphe (5-HT). Distribution of FOSB/ΔFOSB-positive cells was reported on representative drawings adapted from the Paxinos atlas (Paxinos et al., 2001) to illustrate their location and photos (QImaging Retiga 2000R Fast 1394) were taken to illustrate double-labelled cells.

### Statistical analysis

Graph and statistical analyses were performed with GraphPad Prism (GraphPad Software, version 9). Data were presented as the mean ± SD. D’Agostino-Pearson Omnibus normality test was performed to assess the distribution of the data. Comparisons among groups were assessed using Mann-Whitney test. An α-level of 0.05 was used for all tests.

## Results

### Repeated instillations of BLM led to collagen deposition distributed throughout the lung

**FIGURE 2:**
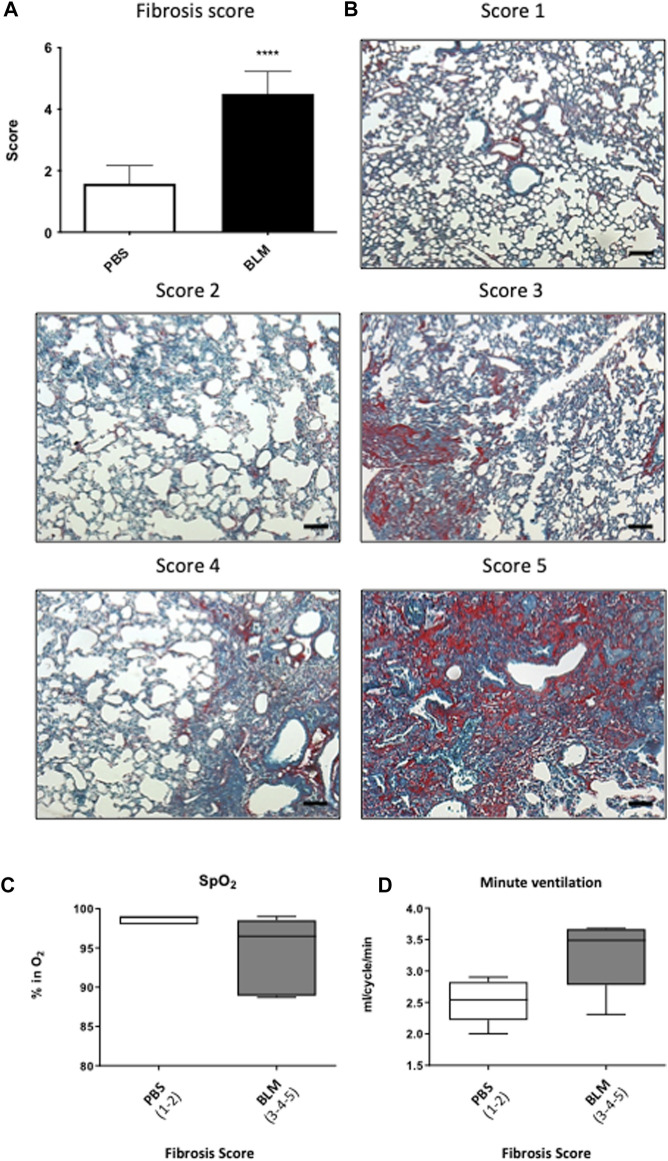
Relationship between the severity of lung damage and ventilatory functions. **(A)** Evaluation of the pulmonary lesion by Fibrosis score. Scores of 1–2 represent less than 20% injury of the total lung area (PBS). Scores between 3 and 5 represent more than 20% injury of the total lung surface (BLM). **(B)** Photographs panel of mouse lungs stained with Sirius red/fast green stain. Score 1 corresponds to a lung with no fibrotic burden at the most flimsy small fibers in some alveolar walls and with normal lung. Score 2 corresponds to clearly fibrotic changes with alveoli partly enlarged and rarefied, but no fibrotic masses. Score 3 corresponds to presence of single fibrotic masses (>10% of microscopic field). Score 4 corresponds to lung with confluent fibrotic masses (>10%) with a lung structure severely damaged but still preserved and score 5 corresponds to large continuous fibrotic masses (>50%) and lung architecture mostly not preserved. **(C,D)** Relationship between **(C)** blood oxygen saturation and **(D)** minute ventilation with the severity of pulmonary fibrosis in PBS (Score 1–2) and BLM (score 3–5) group.

Fifteen days after the first instillation, body weight was significantly lower in BLM group as compared to control (data not shown) and this difference persists until the end (D90) (Table 1). At the lung level, the extent of fibrosis was assessed on whole lung sections stained with Sirius red showing collagen deposition (Figures 1B, C). As expected, a large, compact and extensive collagen deposition with air space reduction was observed in the lung of BLM group as compared to PBS group (Figures 1B, C, 2). Lung fibrosis was then quantified with a score of lesion severity (Figures 2A, B) indicating fibrotic injury. A mean score of 1.6 ± 0.6 (out of 5) was observed in PBS group while a score of 4.5 ± 0.7 (out of 5) was observed in the BLM group (Figures 2A, B).

**TABLE 1:** Body weight and ventilatory variables in PBS (*n* = 6) and BLM (*n* = 6) groups.

		PBS	BLM
Body weight (g)	D0	24.0 ± 1.0	23.0 ± 1.0
	D90	30.5 ± 1.3*	28.2 ±,1.9*
$\dot{V}$ e (ml.g^-1^min^-1^)	D0	2.16 ± 0.13	1.83 ± 0.13
	D90	1.81 ± 0.12	4.81 ± 0.13 ^###^
V_T_ (µL.g^-1^)	D0	8.47 ± 0.61	7.27 ± 0.65
	D90	7.23 ± 1.22	13.87 ± 1.23^###^
*f*_*R*_ (c.min ^-1^)	D0	255 ± 32	255 ± 30
	D90	250 ± 23	346 ± 27 ^*. ###^
T_i_/T_tot_	D0	0.30 ± 0.11	0.29 ± 0.11
	D90	0.31 ± 0.13	0.34 ± 0.11^*. #^
V_T_/T_i_ (µL.g.min^-1^)	D0	0.12 ± 0.17	0.10 ± 0.13
	D90	0.16 ± 0.13	0.41 ± 0.14 ^###^

Values are mean ± standard deviation. * indicates significant difference between D0 and D90 (*p* < 0.05). # indicates significant difference between PBS and BLM group (*p < 0.05, ^#^ *p* < 0.05, ^###^ *p* < 0.001).

### Pulmonary fibrosis has altered the ventilatory mechanics in mice

At the beginning of the protocol (day 0, D0), respiratory variables were not different between PBS and BLM groups (Table 1; Figures 1D, E). By contrast, after 90 days of BLM treatment, minute ventilation ( $\dot{V}$ e) was significantly increased in BLM group as compared to PBS group (Table 1; Figures 1F, G). Indeed, mice with BLM-induced pulmonary fibrosis displayed an important increase in V_T_ and *f*_*R*_ (Table 1; Figures 1E, G). Moreover, at D90, the BLM group displayed a significant increase in the Ti/Ttot and V_T_/Ti ratio as compared to the PBS group (Table 1). Finally, the BLM group showed a significant decrease in lung compliance (Figure 1H) and SpO_2_ (Figure 1I) as compared to the PBS group. Our results showed also that the greater the fibrosis the lower is the SpO_2_ (Figure 2C). Conversely, when the histological fibrosis score increased, $\dot{V}$ e increased (Figure 2D).

### Pulmonary fibrosis induced neuroplasticity at the respiratory network level

**FIGURE 3:**
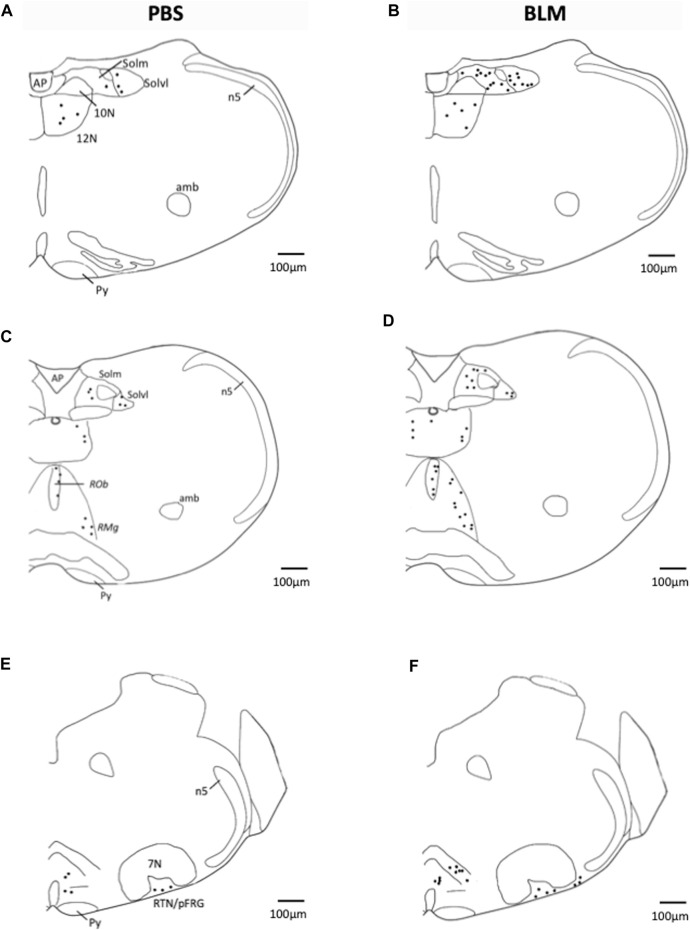
Representative labelling showing FOSB/∆FOSB expression in PBS and BLM condition. Distribution of FOSB/ΔFOSB-positive cells was represented on representative drawings adapted from the Paxinos atlas (Paxinos et al., 2001). **(A–F)** Drawing showing the distribution of the FOSB/∆FOSB positive cells (black dots) in the respiratory related structure in PBS (*n* = 6; **(A,C,E)** and BLM (*n* = 6; **(B,D,F)** groups. Scale bar = 100 µm. Abbreviations:7N: Facial nucleus; 12N: hypoglossal nucleus; Amb: Ambiguus nucleus; AP: Area postrema; n5: Trigeminal nucleus; Py: pyramidal tract; *RMg*: raphe magnus nucleus; *RPa*: raphe pallidus nucleus; *ROb*: raphe obscurus nucleus; RTN/pFRG: retrotrapezoid nucleus/parafacial respiratory group; Solm: median part of nucleus of the solitary tract; Solvl: ventrolateral part of the nucleus of the solitary tract.

**FIGURE 4:**
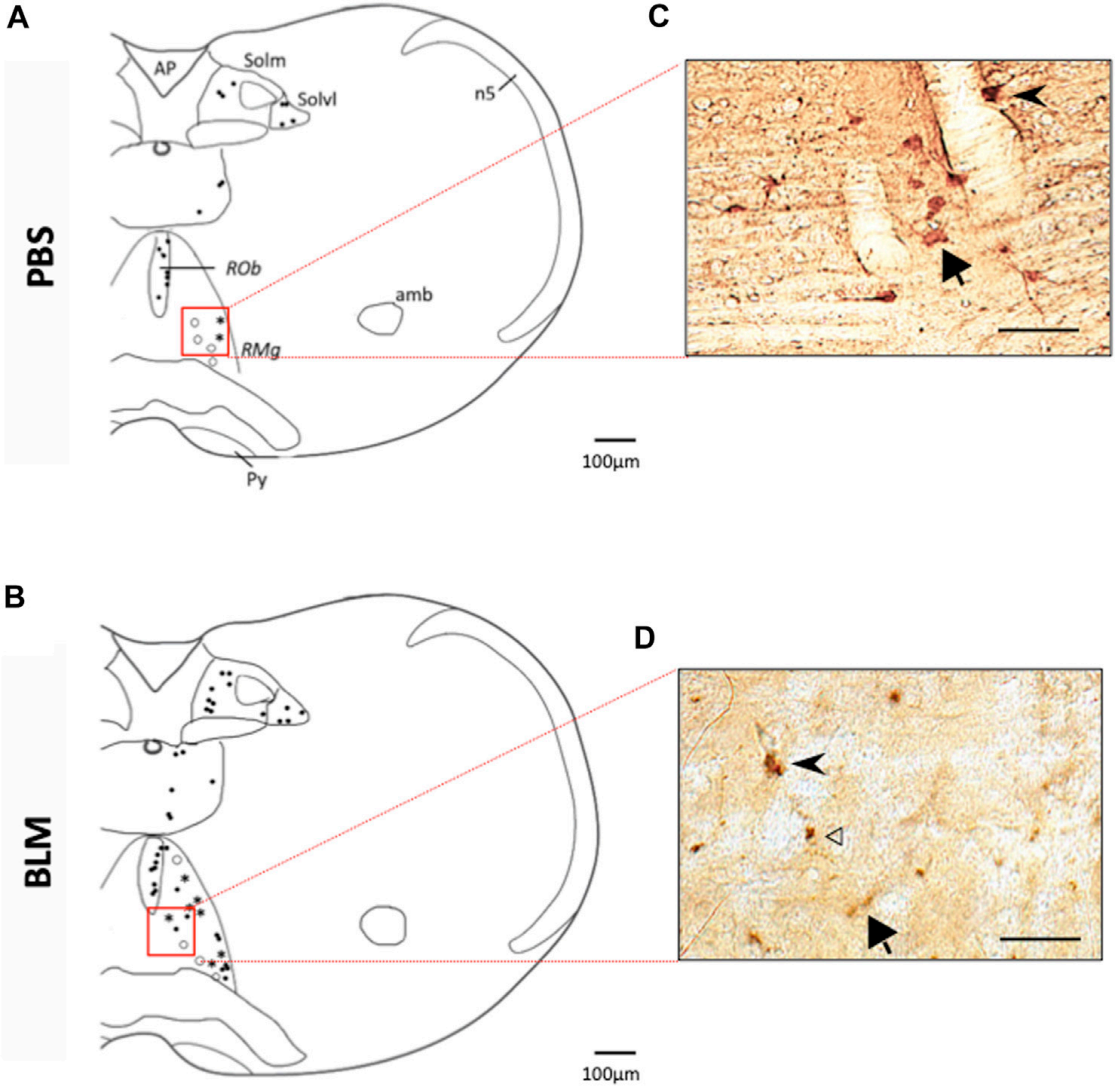
Double labelling of FOSB/ΔFOSB-positive cells with serotonin (5-HT) **(A,B)** Drawing showing the distribution of the FOSB/∆FOSB positive cells (black dots), 5-HT positive cells (White dots), or both (Star) in the Raphe Obscurus (*ROb*) and Raphe Magnus (*RMg*) in PBS **(A)** and BLM **(B)**. Photomicrographs **(C,D)** correspond to the regions delimited in red in the drawings **(A,B)**. White arrowhead indicate FOSB/∆FOSB positive cells, Black arrow indicates 5-HT positive cells and black arrowhead indicate double positive cells. Most of FOSB/∆FOSB positive cells were serotoninergic at the level of the *Raphe magnus*. Scale bar = 100 µm for the drawing and 100 µm for the photos.

**FIGURE 5:**
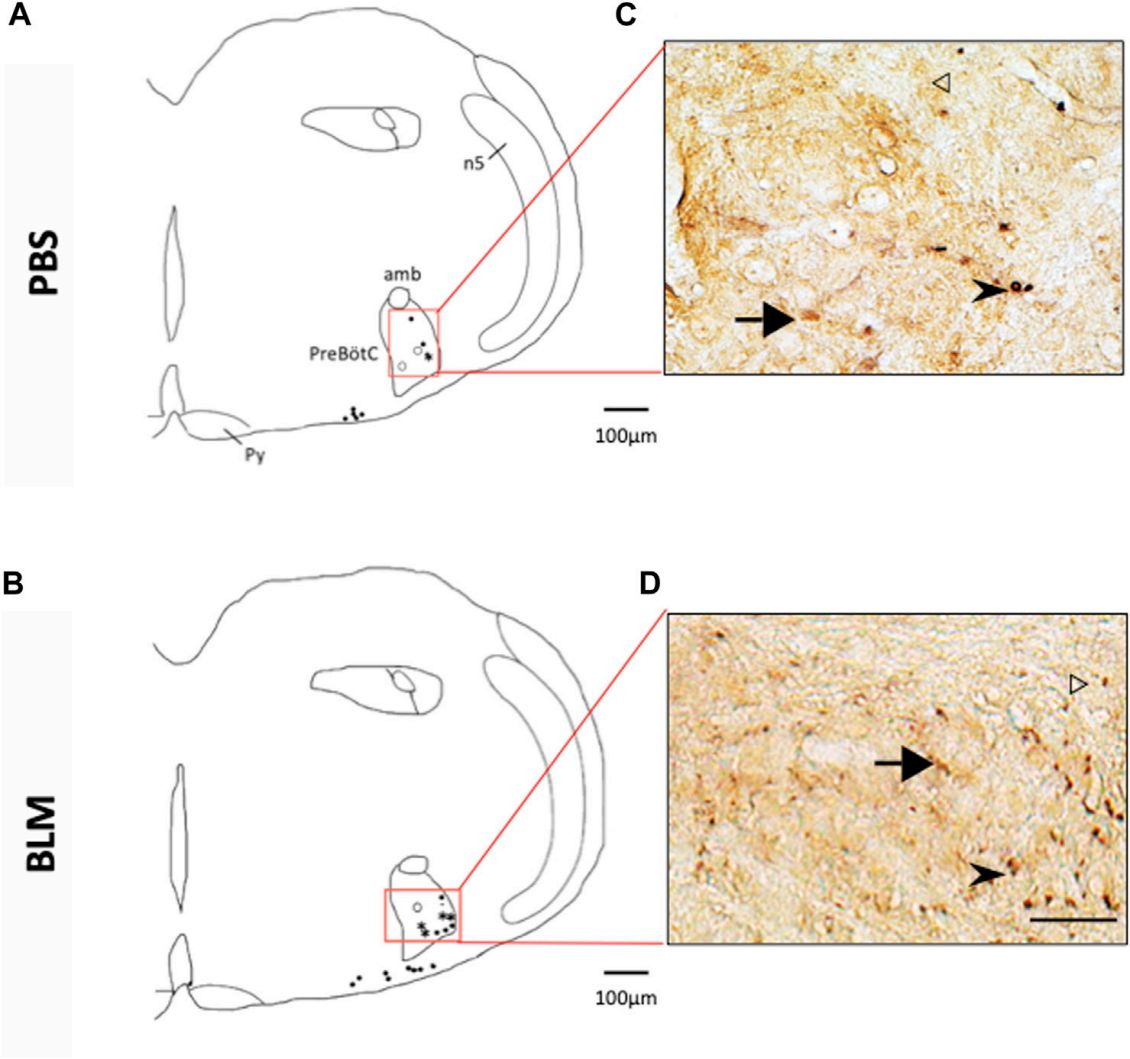
*Double labelling of FOSB/ΔFOSB-positive cells with neurokinin 1 receptor (NK1-R)* **(A,B)** Drawing showing the distribution of the FOSB/∆FOSB positive cells (black dots), NK1-R positive cells (White dots), or both (Star) in the Pre-Bötzinger complex in PBS **(A)** and BLM **(B)** Photomicrographs **(C,D)** correspond to the regions delimited in red on the drawings **(A,B)**. White arrowhead indicate FOSB/∆FOSB positive cells, black arrow indicate NK1-R positive cells and black arrowhead indicate double positive cells. Pulmonary fibrosis-induced FOSB/∆FOSB expression at the level of Pre-Bötzinger complex. Scale bar = 100 µm for the drawing and 100 µm for the photos.

The pulmonary fibrosis-induced neuronal plasticity at the respiratory network level was evaluated through immunodetection of the long-term neuronal markers FOSB/ΔFOSB. As compared to the PBS group, the BLM group displayed a significantly higher number of FOSB/ΔFOSB-positive cells at the level of the first central relay of the peripheral afferents (SolC, +200% and SolM, +180%; Table 2; Figures 3A–D). Some other areas of the central pattern generator increased their long-term activities following induction of pulmonary fibrosis (Table 2), such as SolVL (Figures 3A–D), RTN/pFRG (Figures 3E, F) and RMg (Figures 3C, D), while other areas did not show any change (Table 2). About the RMg, our work shows that 67% of the neurons having modified their long-term activity are serotoninergic (5-HT, Figures 4B, D). NK1-R staining was used to localise more precisely the inspiratory rhythm generator [the Pre-Bötzinger complex (Gray et al., 2001)]. The localisation of the NK1-R distribution associated with that of FOSB/ΔFOSB-positive cells (Figure 5) suggested that neurons of the Pre-Bötzinger complex, the inspiratory rhythm generator, displayed a long-term modification of their activity following an experimental induction of pulmonary fibrosis (Figures 5B, D). Indeed, among the FOSB/ΔFOSB-positive cells in Pre-Bötzinger complex, 100% expressed NK1R in PBS group and 77% expressed it in the BLM group.

**TABLE 2:** Average number of FOSB/ΔFOSB-positive cells in respiratory areas of the medulla oblongata in PBS (*n* = 6) and BLM (*n* = 6) groups.

	PBS	BLM
Solc	5.50 ± 2.16	10.95 ± 6.59**
Solm	5.93 ± 2.37	10.77 ± 2.35*
Solvl	4.54 ± 2.04	7.67 ± 1.75*
RTN/pFRG	5.15 ± 1.77	10.39 ± 5.68**
*ROb*	4.35 ± 1.75	6.60 ± 2.13
*RMg*	5.72 ± 1.56	12.49 ± 5.44**
*RPa*	5.58 ± 1.56	6.96 ± 2.11
12N	5.27 ± 2.53	6.69 ± 2.34

Values are mean ± standard deviation. * indicates significant differences between PBS and BLM group (*p < 0.05, **p < 0.01).

## Discussion

Idiopathic pulmonary fibrosis is a prototypical form of fibrosing interstitial lung disease. This pulmonary pathology of unknown origin is characterized by an excessive accumulation of collagen fibers leading to a thickening of the extracellular matrix (Vuorio et al., 1989). The aim of this study was to characterize the impact of persistent fibrosis on ventilatory function and neuronal plasticity of the ponto-medullary respiratory network. Our main results suggest that BLM-induced pulmonary fibrosis leads to neuronal plasticity.

## Limitation of the study

To interpret our finding, a direct effect of BLM on the CNS must be taken into consideration. Indeed it has been shown that brainstem inflammation modulates the ventilatory pattern and its variability after acute lung injury in rodents (Hsieh et al., 2020). Persistent changes in respiratory patterns following BLM-induced acute lung injury could be due, at least in part, to pro-inflammatory cytokines in the nucleus of the solitary tract (Hsieh et al., 2020). However, in a study designed to investigate the distribution of BLM on mice with methylcholantrene-induced gliomas, BLM given intravenously had a relatively high uptake by the glioma, though little entered the brain tissue (Hayakawa et al., 1974). This study suggested that systematically administrated BLM hardly passes an intact blood brain barrier to enter the CNS (Levin et al., 1979). In our study, BLM was administrated intratracheally, and to our knowledge there is no study that investigated the brain distribution of BLM following this kind of administration. Thus, it is difficult to differentiate a direct central effect of BLM from an indirect effect related to its peripheral action. On the other hand, a unique intratracheal instillation of BLM led to a reduction of the ability of the 2^nd^ order nucleus of the solitary tract neurons to respond to the neurotransmitter release from presynaptic afferents fibers (Getsy et al., 2019) but mechanisms involved are not fully understood. A direct effect of BLM on parietal mechanoreceptors and/or chemoreceptors could also be envisaged. Thus it cannot be excluded that our results may also be related at least in part to a direct effect of BLM. Therefore, to discriminate the effect of pulmonary fibrosis from the effect of BLM, one approach would be to use alternative murine models of pulmonary fibrosis.

Neuroplasticity is the biological, chemical, and physical capacity of the brain to reorganize its structure and function. Thus, neuroplasticity could be evaluated through different ways. For example, *in vivo* Magnetic Resonance Imaging (MRI) could be an interesting approach, at least in humans, in order to extract functional, structural and biochemical information from the entire brain and identify regions who are more or less active (Hamaide et al., 2016). However, the use of this technique is more confidential in animals and requires other approaches such as evolution of the phrenic activity (MacFarlane et al., 2018), patch-clamp technique (Kida et al., 2017), histological analysis (Baum et al., 2018), etc. Histological analysis of the expression of transcription factors belonging to Activator Proteins 1 (AP-1) complexes is a common experimental approach to evaluate respiratory neuroplasticity. AP-1s are composed of homo- or heterodimers of JUN and FOS, inducible Leucine Zippers-type transcription factors. The proteins c-FOS and FOSB are expressed at a low basal level in the central nervous system and accumulate during a stimulus, thus constituting neuroplasticity markers of cellular response (Herdegen et al., 1991; Herdegen and Leah, 1998; Nestler et al., 2001). Neuroplasticity can be induced by several mechanisms which generate short- and long-term potentiation (STP and LTP) through a cascade of reactions. While STP mainly involves already synthesized signaling factors such as kinases, LTP requires *de novo* biosynthesis via transcription factors such as FOSB/ΔFOSB. Moreover, we previously examined long-term changes in the activity of brainstem cardiorespiratory network induced by chronic intermittent hypoxia (CIH) in mice (Baum et al., 2018). In this study, we demonstrated that 21 consecutive days of intermittent hypoxia was sufficient to induce neuronal plasticity. Furthermore, it was also demonstrated that 7 days of intermittent hypoxia chronically increases blood pressure and that this increase is accompanied by an expression of FOSB/ΔFOSB in central cardiorespiratory structures involved in sympathetic activity (Knight et al., 2011). Thus we assume that our experimental protocol is long enough to evaluate functional effects of pulmonary fibrosis to the brainstem respiratory network.

### Pulmonary fibrosis alters ventilatory mechanics

**FIGURE 6:**
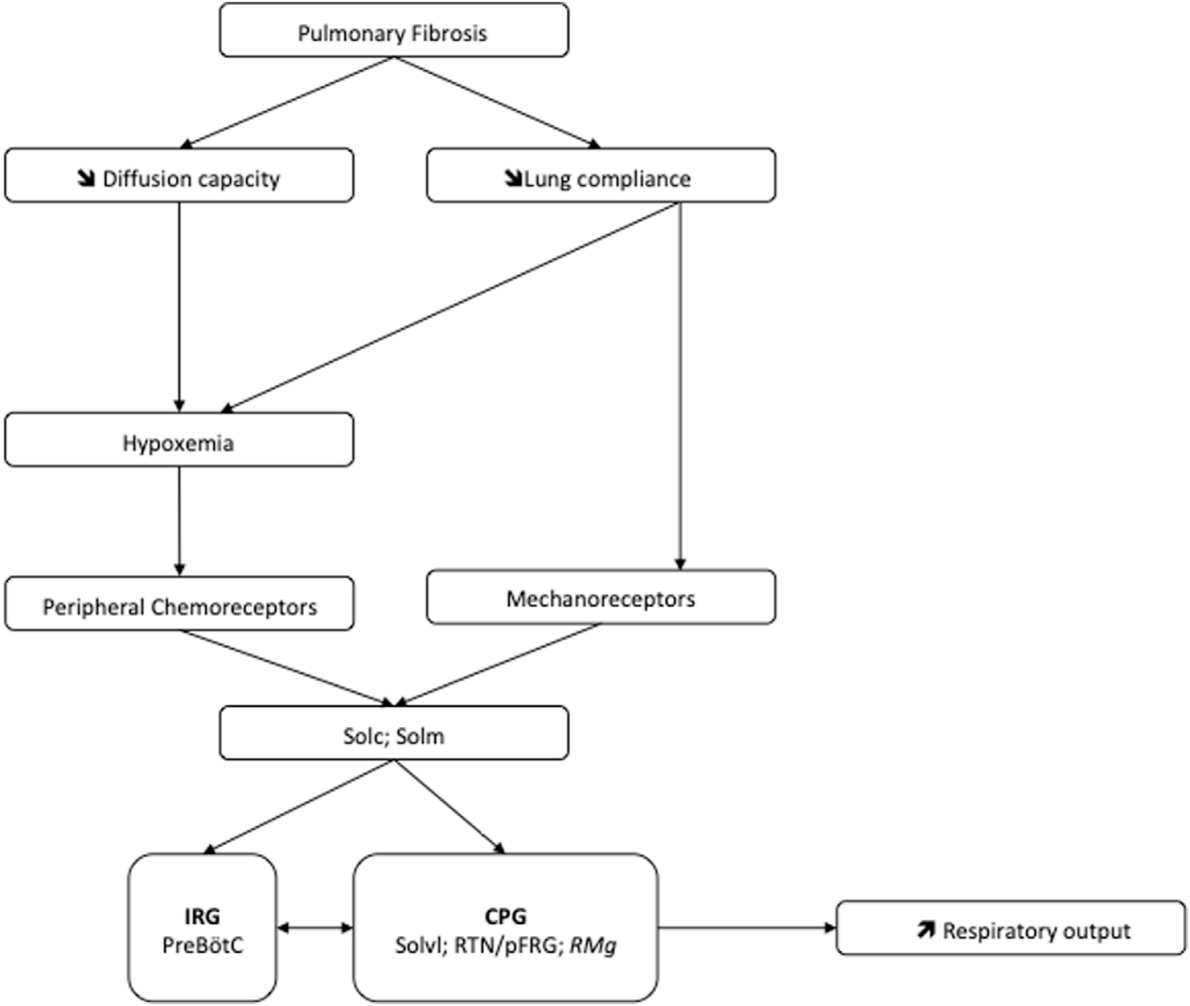
Potential mechanism at the origin of the pulmonary fibrosis-induced neuroplasticity leading to increase of the ventilatory drive. Abbreviations: CPG, Central Pattern Generator; IRG, Inspiratory rhythm generator; PreBötC, Pre-Bötzinger complex; *RMg:* raphe magnus nucleus; RTN/pFRG, retrotrapezoid nucleus/parafacial respiratory group; Solc, commissural part of nucleus of the solitary tract; Solm, median part of nucleus of the solitary tract; Solvl, ventrolateral part of the nucleus of the solitary tract.

We observed alteration of ventilatory variables in our mouse model of chronic lung fibrosis, which reminds us, at least in part, what is observed in IPF patients (Olukogbon et al., 2016). These patients display an increase in minute ventilation associated with increased respiratory rate and decreased tidal volume (Javaheri and Sicilian, 1992; Fumeaux et al., 2003). Indeed, in patients, an impairment of ventilatory variables has been observed, characterized by a low value of forced vital capacity associated with an increase in *f*_R_, and a decrease in V_T_ (Javaheri and Sicilian, 1992). In contrast, mice with BLM-induced pulmonary fibrosis displayed a large increase in V_T_ and *f*_R_. However, the absolute value of V_T_ and the minute ventilation derived from Drorbaugh and Fenn equation (Drorbaugh and Fenn, 1955) are indicative while temporal measurements such as *f*_R_ are reliable. Indeed, the whole body plethysmography is a barometric plethymography method. For this, mice were placed in subject chamber whereas reference chamber remains empty. The pressure in the subject chamber varies with the subject’s breathing. Each of the subject’s breaths creates two distinct but related signal: a pressure modification generated by flow of air into and out of the nose and pressure modification generated by compression/expansion of air when the thorax rises and falls. Thus, results given by plethysmography approach reflect the functioning of the respiratory system as a whole (thoraco-pulmonary system, airways.) and did not only reflect V_T_ unlike that observed in patients where V_T_ was measured directly via a mouthpiece and reflects the volumes entering and leaving the airways. Thus, the observed increase in V_T_ may reflect an increase in respiratory effort in the face of decreased compliance. Indeed, the pressure oscillation was affected not only by V_T_ of the mechanical chest but also by *f*_R_ and by airway resistance (Enhorning et al., 1998). Moreover, inflammation is recognized as a mediators affecting control of breathing (Andrade et al., 2018). Furthermore, inflammation undermines neuroplasticity, including serotonin-dependent phrenic long-term facilitation (pLTF) following moderate acute intermittent hypoxia (Marciante and Mitchell, 2023). However, our model has the advantage of inducing a persistent lung fibrosis without major inflammation, reproducing histology features observed in UIP (Yegen et al., 2022) (Yegen et al., 2022). Although this remains to be verified, we assume that there is no inflammation in the CNS either, and therefore the observed increase in $\dot{V}$ e is probably not related to this. Similarly, the breathing time as well as an index of the inspiratory drive (given respectively by The Ti/Ttot and V_T_/Ti ratios; (Milic-Emili and Grunstein, 1976)) was also increased following the induction of pulmonary fibrosis in our model. The increase in the V_T_/Ti ratio was an indication of increased ventilatory drive. In mice, the functional decline has been positively correlated with the degree of pulmonary fibrosis (Manali et al., 2011) and the work of breathing was shown to be significantly correlated with lung fibrosis histopathology score (Phillips et al., 2012), in line with our results. Our murine model of pulmonary fibrosis obtained after repeated BLM instillations presents a diffuse collagen deposition in the whole lung (Yegen et al., 2022). This could explain, at least in part, the decrease in pulmonary compliance. This decrease is likely the consequence of increased stiffness of the pulmonary parenchyma and is consistent with that observed in the literature for murine models of pulmonary fibrosis obtained by repeated instillations (Redente et al., 2021). As there are mechanical afferents from mechanoreceptors located in the lung parenchyma, bronchi and respiratory muscles (Raux et al., 2007), the modification of lung stiffness could affect these thoraco-pulmonary mechanoreceptors, which in turn can affect the control of ventilation (Figure 6). Indeed, a disturbance of ventilatory mechanics can have a significant effect on the respiratory control (Maszczyk et al., 1990). Furthermore, we observed that mice with pulmonary fibrosis, 90 days after the first intratracheal instillation of BLM, showed a decrease in SpO_2_, as observed in patients with severe IPF (Stephan et al., 2007; Takei et al., 2020). The observed desaturation could activate peripheral chemoreceptors (Khoo et al., 1982; Lahiri et al., 1983) and participate in the augmentation of $\dot{V}$ e (Figure 6). Thus, modifications of ventilatory variables in our model could be the consequence of either altered pulmonary mechanics and/or altered gas exchanges at the level of the alveolar-capillary membrane (Figure 6). The pulmonary fibrosis-induced increase in ventilation could reflect the need for the animals to make a ventilatory “effort” to compensate the increase in pulmonary stiffness and/or could be related to the presence hypoxemia resulting from the alteration of gas exchange through the thickening of the alveolar-capillary membrane (Launois et al., 1991).

### Pulmonary fibrosis led to a neuroplasticity phenomenon

The mechanism of breathing results from the rhythmic contraction of the ventilatory muscles, which depends on the central respiratory drive that determines *f*_R_ and V_T_. A neuronal network located at the ponto-medullary level generates this central command and receives different kinds of afferents including metabolic afferents from central and peripheral chemoreceptors (Raux et al., 2007; Dean and Putnam, 2010). The variations in ventilatory variables observed in our mouse model of pulmonary fibrosis could also reflect a change in the functioning of this respiratory neuronal network. Our hypothesis was that the modification of the pulmonary mechanics and/or hypoxemia due to fibrosis could be at the origin of a neuroplasticity. Neuroplasticity can be divided into two major mechanisms: neuronal regeneration/collateral sprouting and functional reorganization. More precisely, the respiratory neuroplasticity describes “the persistence of morphological and/or functional neuronal changes following previous experiences” (Mitchell and Johnson, 2003). Neuroplasticity could be induced by several mechanisms such as the reactivation axon terminals, changes in the synthesis, release or re-uptake of neurotransmitters/neuromodulators, a modification in the excitability of neurons by changes in the membrane potential or the formation of new nerve connections. As mentioned above, these mechanisms generate STP and LTP through a cascade of reactions which can lead to a modification of the neuronal network, the stability of a synaptic connection, and/or the number of axon terminals that stimulate a dendrite (Fuller and Mitchell, 2017). Thus, we hypothesize that modification of lung architecture due to pulmonary fibrosis can lead to chronic stimuli through peripheral chemoreceptors and/or mechanoreceptors, which are likely to induce long-lasting functional changes in the central respiratory drive.

In IPF patients, the increase in ventilatory effort induced by exercise could be attributed to increased afferents from the lungs and/or chest wall (Van Meerhaeghe et al., 1981). Furthermore, the disturbance of the ventilatory mechanics can have a significant effect on the respiratory control (Maszczyk et al., 1990). In our mouse model of pulmonary fibrosis, analysis of the various structures within the respiratory neural network revealed an increase in the number of FOSB/∆FOSB positive cells suggesting that some of them modify their activities in response to lung damage. Indeed, we assessed the neuroplasticity by immunodetection of the long-term neuronal markers FOSB/∆FOSB (Baum et al., 2018). In this latter study, as in our study, it was shown that chronic intermittent hypoxia induced modification of FOSB/∆FOSB at the level of SolC and rVLM (Baum et al., 2018). Modification of Sol activity contributes to both the development and maintenance of plasticity (Ostrowski et al., 2023). Indeed, SolC neurons received primary afferent from the lungs, carotid body, etc. and are able to modulate respiratory response through connections with different brain region. Among these regions there are respiratory-related areas of the medulla, such as the rVLM, the RTN/pFRG (Otake et al., 1992; Accorsi-Mendonça et al., 2011) via excitatory transmission (Accorsi-Mendonça et al., 2011; Lima-Silveira et al., 2019). Thus, our results suggested that the thoraco-pulmonary mechanoreceptors and/or the chemoreceptors, via their afferents, could be at the origin of a neuroplasticity phenomenon affecting respiratory control. It was previously suggested that the ventilatory alterations observed in patients with stable diffuse interstitial lung disease would be the result of neural mechanisms (Gaultier, 1983). Our results tried to elucidate this point. It seems that lung damage could be associated with long-term changes in the sensitivity of pulmonary sensors (Undem, 2005) and/or altered synaptic plasticity in second-order neurons in the nucleus of the solitary tract as well as in higher-order synapses (Getsy et al., 2019). Indeed, SolC and SolM are the first central relays of information from the peripheral chemosensory and mechanical afferents (Torrealba and Claps, 1988). Neurons in the SolC and SolM integrate and relay information to the respiratory neuronal network including PreBötC, RTN/pFRG or Raphe Nuclei (Takakura et al., 2006; Smith et al., 2013). Therefore, changes in chemo and/or mechanoreceptors activity can affect the entire respiratory neuronal network and explain the observed increase at the level of the PreBötC and CPG structures leading to an increase of the respiratory motor output, which could modify the respiratory pattern (Figure 6). Thus, it seems that chest wall mechanoreceptors sensitive to rib cage expansion, as well as chemoreceptors, could contribute to these responses. Other experiences would be useful to differentiate the involvement of each mechanism. Furthermore, pulmonary fibrosis induced an increase in the number of FOSB/∆FOSB positive cells at the level of the RTN/pFRG and the 5-HT neurons of RMg, two areas involved in central chemosensitivity (Dias et al., 2007; Guyenet and Bayliss, 2015). These results suggested that the decrease in the ventilatory response to hypercapnia reported in patients with pulmonary fibrosis (Launois et al., 1991) could have a central origin. This point requires further experiments. Moreover, serotoninergic systems are known to be involved in many processes of ventilatory neuroplasticity, making 5-HT one of the chemical messengers of neuroplasticity. Thus our results suggest a form of neuroplasticity in the respiratory neuronal network.

### Clinical and scientific significance of the study

Patient with IPF are disabled by dyspnea, which impairs their quality of life (Martinez et al., 2017; Plantier et al., 2018). Decrease in lung compliance, hypoxemia, increase in dead space ventilation or increase of work of breathing have all been widely described as being involved in dyspnea. However the role of these factors as a primary determinant of dyspnea is unclear. The dyspnea could also result from a mismatch between the respiratory motor output developed by the neuronal respiratory network and the accomplished ventilatory motor activity (Fukushi and Okada, 2019; Fukushi et al., 2021). Indeed, higher brain centers compare the respiratory motor command corollary discharge to the information coming from lung mechanoreceptors, and dissociation between both signals may be at the origin of dyspnea. Thus an alteration of the functioning of the respiratory neural network as presented in this paper could participate and reinforce this feeling of respiratory discomfort. Improving ventilation by a pharmacological impact on the respiratory neural network could break the vicious circle of dyspnea and could be a way to improve the quality of life of patients. Further studies will be necessary to explore this possibility.

Another important point would be to know what is the contribution of the mechanoreceptors *versus* chemoreceptors in the observed responses. Furthermore, it would be necessary to be able to distinguish the sites of projections of the mechanoreceptors from those of the chemoreceptors. Indeed, we suggested that the thoraco-pulmonary mechanoreceptors and/or the chemoreceptors, via their afferents, could be at the origin of a neuroplasticity phenomenon affecting respiratory control. Thus in not-hypoxemic patient, respiratory activity could be affected only by mechanical aspect of architectural modification. Indeed, we could hypothesize that inputs from the mechanical receptors are predominant signals for respiratory alterations in mild fibrosis whereas those from chemical receptors further augments respiration in severe fibrosis. Further studies will be necessary to confirm this hypothesis.

## Conclusion

Our results show that pulmonary fibrosis-induced lung damages affect the central respiratory drive through their consequences on ventilatory mechanics and/or gas exchange. This effect could lead to an increase in the command to the respiratory muscles. The activation of the respiratory neurons in response to this pulmonary damage, suggested that the observed ventilatory anomalies could be not only related to peripheral lung mechanical effects but also probably to a central adaptation of the respiratory neural network (Figure 6).

## Data Availability

The original contributions presented in the study are included in the article/supplementary material, further inquiries can be directed to the corresponding authors.
